# Supplementary material for: Metagenomic binning with assembly graph embeddings
Source: Bioinformatics. 2022 Aug 16;38(19):4481–7. doi: 10.1093/bioinformatics/btac557 (PMC9525014; doi:10.1093/bioinformatics/btac557)
Supplement: btac557_supplementary_data [file btac557_supplementary_data.zip › amber outputs.zip!/amber outputs/simHC/genome/metabat_edges/heatmap_simHC+.pdf]

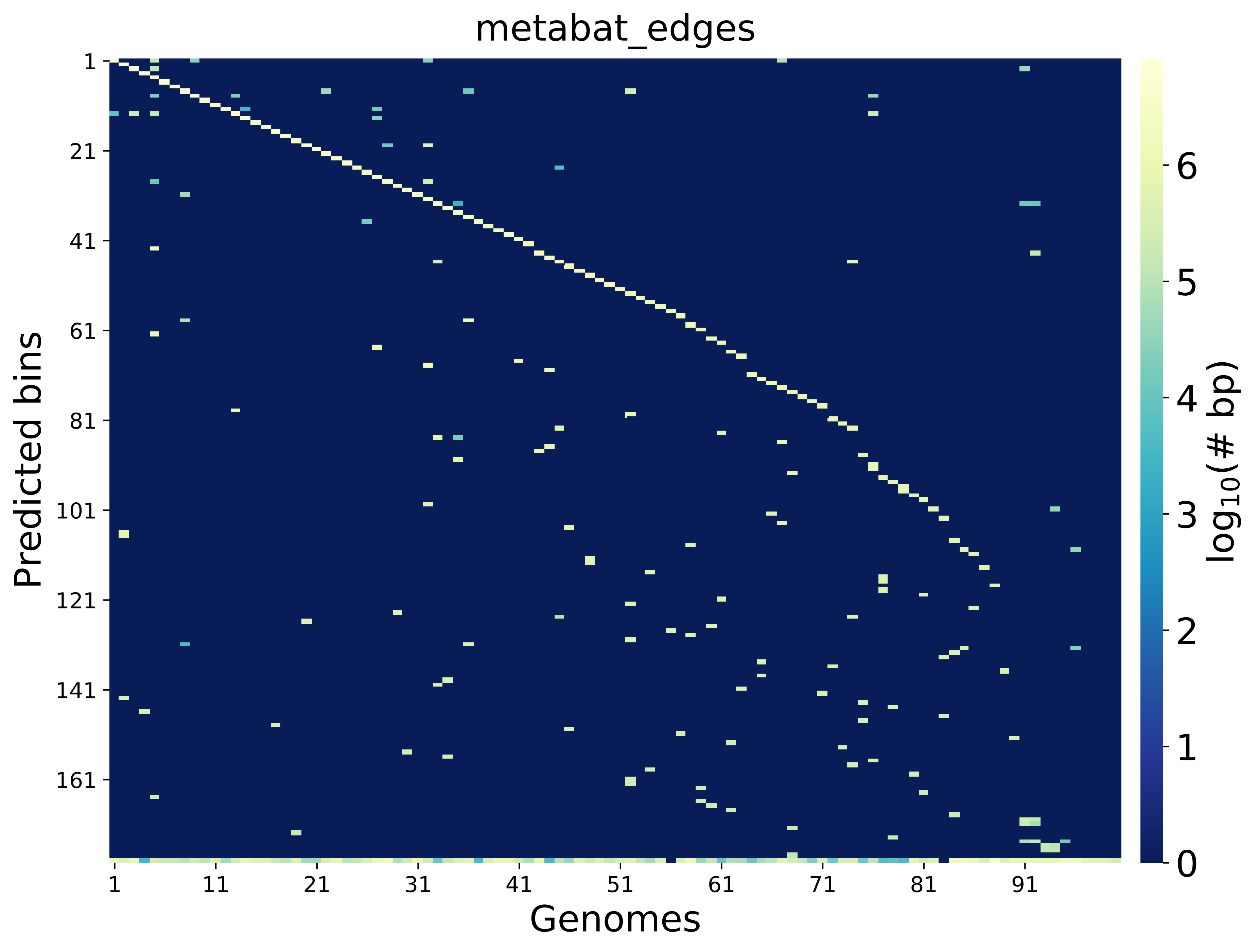
metabat_edges
Predicted bins
1
21
41
61
81
101
121
141
161
Genomes
1
11
21
31
41
51
61
71
81
91
log10(# bp)
0
1
2
3
4
5
6
